# Characterising the biology of novel lytic bacteriophages infecting multidrug resistant *Klebsiella pneumoniae*

**DOI:** 10.1186/1743-422X-10-100

**Published:** 2013-03-28

**Authors:** Agata Kęsik-Szeloch, Zuzanna Drulis-Kawa, Beata Weber-Dąbrowska, Jerzy Kassner, Grażyna Majkowska-Skrobek, Daria Augustyniak, Marzanna Łusiak-Szelachowska, Maciej Żaczek, Andrzej Górski, Andrew M Kropinski

**Affiliations:** 1 Institute of Genetics and Microbiology, University of Wroclaw, Przybyszewskiego 63/77, Wroclaw, 51-148, Poland; 2 L. Hirszfeld Institute of Immunology and Experimental Therapy, Polish Academy of Sciences, Centre of Excellence, Weigla 12, Wroclaw, 53-114, Poland; 3 Department of Clinical Immunology, The Medical University of Warsaw, Nowogrodzka 59, Warszawa, 02-006, Poland; 4 Laboratory for Foodborne Zoonoses, Public Health Agency of Canada, 110 Stone Road West, Guelph, ON, N1G 3 W4, Canada; 5 Department of Molecular & Cellular Biology, University of Guelph, 50 Stone Road East, Guelph, ON, N1G 2 W1, Canada

**Keywords:** Bacteriophage, *Klebsiella* spp., Multidrug resistance, Restriction endonuclease patterns, *Myoviridae*, *Siphoviridae*, *Podoviridae*

## Abstract

**Background:**

Members of the genus *Klebsiella* are among the leading microbial pathogens associated with nosocomial infection. The increased incidence of antimicrobial resistance in these species has propelled the need for alternate/combination therapeutic regimens to aid clinical treatment. Bacteriophage therapy forms one of these alternate strategies.

**Methods:**

Electron microscopy, burst size, host range, sensitivity of phage particles to temperature, chloroform, pH, and restriction digestion of phage DNA were used to characterize *Klebsiella* phages.

**Results and conclusions:**

Of the 32 isolated phages eight belonged to the family *Myoviridae*, eight to the *Siphoviridae* whilst the remaining 16 belonged to the *Podoviridae*. The host range of these phages was characterised against 254 clinical *Enterobacteriaceae* strains including multidrug resistant *Klebsiella* isolates producing extended-spectrum beta-lactamases (ESBLs). Based on their lytic potential, six of the phages were further characterised for burst size, physicochemical properties and sensitivity to restriction endonuclease digestion. In addition, five were fully sequenced. Multiple phage-encoded host resistance mechanisms were identified. The *Siphoviridae* phage genomes (KP16 and KP36) contained low numbers of host restriction sites similar to the strategy found in T7-like phages (KP32). In addition, phage KP36 encoded its own DNA adenine methyltransferase. The φKMV-like KP34 phage was sensitive to all endonucleases used in this study. Dam methylation of KP34 DNA was detected although this was in the absence of an identifiable phage encoded methyltransferase. The *Myoviridae* phages KP15 and KP27 both carried Dam and Dcm methyltransferase genes and other anti-restriction mechanisms elucidated in previous studies. No other anti-restriction mechanisms were found, e.g. atypical nucleotides (hmC or glucosyl hmC), although *Myoviridae* phage KP27 encodes an unknown anti-restriction mechanism that needs further investigation.

## Background

Bacteriophages, or phages, are viruses that infect bacteria. They are the most abundant and the most genetically diverse biological entities on Earth, with global numbers estimated at 10^30^ to 10^32^[1,2]. These viruses are ubiquitous throughout the environment and are found in all environments that support bacterial proliferation [3,4]. It is now realized that phages play an important role in the cycling of organic matter in the biosphere and play a significant role in bacterial diversity [5,6]. Successful infection by lytic bacteriophages requires attachment to a susceptible host cell through specific binding to a surface epitope, injection of the phage nucleic acid, replication, virion assembly and, finally, release of infectious progeny [7]. In the case of Gram-negative bacteria phage adsorption to specific receptors such as pili, flagella, capsules, outer membrane proteins and lipopolysaccharides has been demonstrated [8].

The specificity of interaction between phage tail structures and host receptor defines the host range of these viruses [9]. Some phages have specificity at the strain level whereas some have broader host ranges and can infect multiple bacterial strains within a single species or even multiple related species [10]. To achieve successful infection and propagation, bacteriophages need to overcome the host resistance mechanisms that target foreign DNA on entry into the bacterium. Four types of resistance mechanisms have been categorised through their mode of action and how they target the phage life cycle. These include: adsorption inhibition, blocking of DNA injection, restriction-modification (RM) and abortive infection [11]. Arguably, the most well-studied anti-phage defence mechanism is the restriction-modification system, which is present in over 90% of sequenced bacterial genomes [12]. There are several antirestriction mechanisms developed by bacteriophages [13,14]: (i) counter selection against relevant restriction sites in phage genome exemplified by coliphage T7; (ii) inhibitors of host restriction enzyme – Ocr proteins of phages T7 and T3 block type I and type III of RM systems; (iii) hydrolysis of RM system cofactors – e.g. hydrolysis of S-adenosylmethionine by T3 resulting in blockage of type I and type III RM systems; (iv) co-injection of DNA and RM inhibitors – the DarA and DarB proteins of P1 block the type I RM system; (v) stimulation of host methyltransferase activity (type I system) – Ral and Lar proteins of λ-like phages; (vi) DNA modifying enzymes – acquisition of Dam and Dcm methyltransferase genes by T4-like phages; (vii) incorporation of hypermodified nucleotides into DNA – hydroxymethylcytosine (hmC) or glucosylated hydroxymethylcytosine (glucosyl hmC) in T4-like phages [15].

Bacteriophages have been of interest to scientists as tools to understand fundamental molecular biology processes, as vectors for horizontal gene transfer and drivers of bacterial evolution. Moreover, bacterial viruses are convenient sources of diagnostic and genetic tools and have the potential to be used as novel therapeutic agents [4,16]. With the increased incidence of multidrug resistance in bacteria, therapeutic and preventive options have become limited. One of the possible alternatives to antibiotics is the application of bacteriophages or phage proteins. The idea/implementation of using phages as a therapeutic intervention is well known. As antimicrobial drugs entering the pharmaceutical market is limited, there is a need for novel therapeutics where phages with lytic potential and “generally regarded as safe” (GRAS) status by the FDA fall into this category. Multidrug resistant *Klebsiella pneumoniae* isolates carrying extended-spectrum beta-lactamases (ESBLs) encoding plasmids are becoming increasingly associated with nosocomial infection. At present the prevalence of ESBL-producing *Klebsiella* strains in Europe has reached 10-30% of invasive isolates [17]. Antibiotic usage in clinical settings, and also in animal husbandry, has led to the maintenance of ESBL-encoding bacteria in the environment [18]. It is now well documented that ESBL-producing bacteria may also have a zoonotic origin with strains isolated from poultry, a pig farm and retail meat [19-21]. The high incidence of multidrug resistant bacteria has resulted in limited efficacy of treatment with current antibiotics, and a high probability of patient colonization by resistant strains. This manuscript focuses on *Klebsiella* phages and their potential as alternative antimicrobials. Phages possessing a broad spectrum of activity, and belonging to all families within the *Caudovirales*, were isolated and characterized. The main differences detailed in this study are the viral mechanisms for resistance to host restriction systems.

## Results

### Origin and isolation of bacteriophages

**Figure 1:**
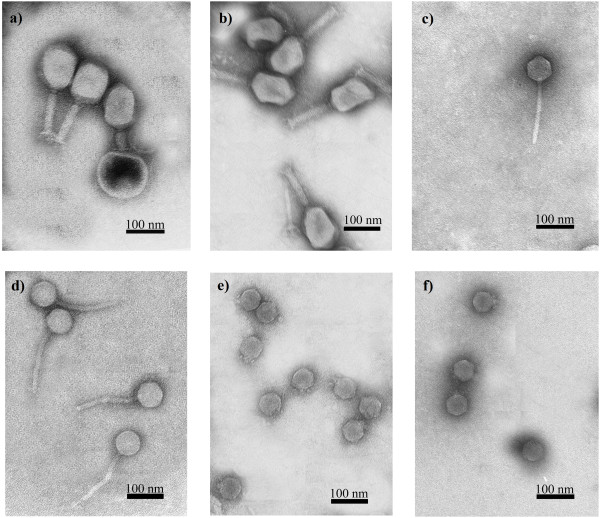
**Transmission electron micrograph of negatively stained phages. a**) KP15 *Myoviridae* family, **b**) KP27 *Myoviridae* family, **c**) KP16 *Siphoviridae* family, **d**) KP36 *Siphoviridae* family, **e**) KP32 *Podoviridae* family, **f**) KP34 *Podoviridae* family. The bar indicates 100 nm.

Eight aquatic samples were screened for the presence of phages (Table 1). The samples taken from a roadside ditch and an excavation pond did not contain somatic coliphages (SOMCPH, infecting the hosts via receptors located on the cell surface) or male-specific (F^+^) coliphages (FRNAPH, infecting the “male” bacteria via the sex pili), which may be interpreted as no/low contamination with enteric bacteria. Using 11 ESBL*-*producing clinical *K. pneumoniae* strains as the host, 32 lytic bacteriophages were propagated (Table 1). The majority of phages were found in sewage samples collected from a natural waste-water treatment plant (irrigated fields) located in Wrocław, Poland. The coliform bacteria contamination of irrigated fields was very high, as indicated by the titres of the phage indicators: 4 × 10^4^ PFU/100 ml and 5 × 10^4^ PFU/100 ml for SOMCPH and FRNAPH, respectively. All the isolated bacteriophages were examined by transmission electron microscopy and classified on the basis of their morphological features in the order *Caudovirales* and its virus families: *Myoviridae* (eight isolates, T4-like), *Siphoviridae* (eight isolates) and *Podoviridae* (16 isolates) (Figure 1). The isolates were named according to the newly proposed naming system vB KpnP/M/S KPno, where vB = bacterial virus; Kpn = REBASE abbreviation for genus/species of the host; P = podovirus, M = myovirus, S = siphovirus; KPno = name and number of phage [22]. All the phages from the *Myoviridae* family produced 1 mm clear plaques, while members of the *Siphoviridae* produced plaques approximately 3–5 mm in diameter surrounded by a large halo. The plaques of members of the *Podoviridae* family were generally approximately 3 mm in size, surrounded by a large halo, the exception being phage KP32, which produced large (5–7 mm) clear plaques with small halos. Plaque morphology of all phages was determined using the same medium and conditions; thus the differences resulted from the properties of each phage.

**Table 1:** Water samples used as a bacteriophage source

**Sample**	**Origin**	**SOMCPH/FRNAPH titre (PFU/100 ml)**	**Number of isolated phages (No)**
Mechanical treated sewage	Communal Wastewater Treatment Plant (Stronie Śląskie, Poland)	1 × 10^2^/3,4 × 10^2^	2 phages (23, 31)
Biological treated sewage	Communal Wastewater Treatment Plant (Stronie Śląskie, Poland)	4 × 10^1^/4,4 × 10^2^	3 phages (9, 27, 36)
Sewage sample	Irrigated fields 1 (Wrocław, Poland)	4 × 10^4^/5 × 10^4^	6 phages (1, 2, 4, 8, 24, 26)
Sewage sample	Irrigated fields 2 (Wrocław, Poland)	2 × 10^1^/4 × 10^1^	11 phages (6, 10, 14, 15, 17, 18, 19, 20, 21, 22, 29)
Sewage sample	Cesspool holding tank (Nieciszów, Poland)	0/2 × 10^1^	4 phages (12, 13, 28, 34)
Environmental water	Roadside ditch (Nieciszów, Poland)	4 × 10^1^/2 × 10^1^	4 phages (5, 7, 16, 32)
Environmental water	Home well (Strachocin, Wrocław, Poland)	0/0	1 phage (25)
Environmental water	Excavation pond “Biała Marianna” Sienna (Poland)	0/0	1 phage (33)

### Determination of phage host range

**Figure 2:**
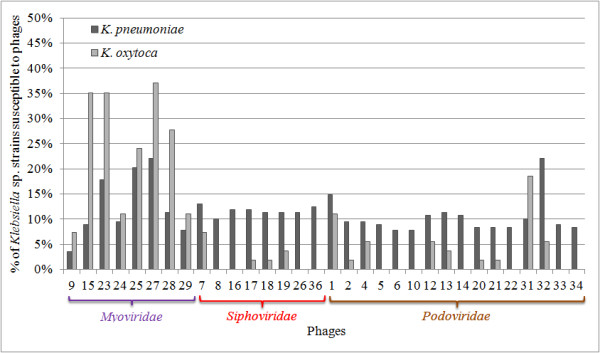
**Activity of KP bacteriophages against *Klebsiella *sp. isolates listed in Table**2**.**

The lytic activity of isolated viruses was examined on 254 bacterial strains (Table 2). All of the *Pantoea* spp., *Enterobacter* spp., *Escherichia coli*, *Klebsiella ozaenae*, *Klebsiella mobilis* and *Klebsiella rhinoscleromatis* tested strains were found to be resistant to these phages. Members of the *Siphoviridae* and *Podoviridae* exhibited generally similar activity against *Klebsiella pneumoniae* strains lysing 7-15%. The exception was phage KP32, which lysed 22% (Figure 2). Only 13 of these 24 phages propagated on *Klebsiella oxytoca* strains. Generally members of the *Myoviridae* exhibited higher lytic activity against *K. oxytoca* strains (7-37%), even if they were propagated on *K. pneumoniae* as a host. There was no correlation found between bacterial ESBL enzyme production and susceptibility to particular phage.

**Table 2:** **Bacterial strains used for determination of *Klebsiella *phages host range**

**Strain**	**Number of isolates**	**Reference**
*Klebsiella pneumoniae* subsp. *pneumoniae* ESBL(+)*	1	ATCC 700603
*Klebsiella pneumoniae* subsp. *pneumoniae*	96	Clinical isolates; own collection
*Klebsiella pneumoniae* subsp. *ozaenae*	2	PCM 4; PCM 6**
*Klebsiella pneumoniae* subsp. *rhinoscleromatis*	2	PCM 2066; PCM 2067**
*Klebsiella oxytoca*	48	Clinical isolates; own collection
*Klebsiella mobilis (Enterobacter aerogenes)*	3	Clinical isolates; own collection
*Enterobacter cloacae*	50	Clinical isolates; own collection
*Pantoea (Enterobacter) agglomerans*	1	Clinical isolate; own collection
*Cronobacter* (*Enterobacter*) *sakazakii*	1	Clinical isolate; own collection
*Escherichia coli*	50	Clinical isolates; own collection

*ESBL – extended-spectrum beta-lactamase producing strain (genes located on plasmid).

**PCM – Polish Collection of Microorganisms (Institute of Immunology and Experimental Therapy, Polish Academy of Sciences, Weigla 12, 53–114 Wrocław, Poland).

For further detailed biological characterization six phage isolates were chosen: (i) vB_KpnM_KP15 from an irrigated field and vB_KpnM_KP27 from a communal wastewater treatment as *Myoviridae* representatives; (ii) vB_KpnS_KP16 from a roadside ditch and vB_KpnS_KP36 from a communal wastewater treatment as *Siphoviridae* representatives; (iii) vB_KpnP_KP32 from a roadside ditch and vB_KpnP_KP34 from a cesspool as *Podoviridae* representatives.

### Lytic potential and physicochemical properties of selected bacteriophages

A high percentage (98.5-99.7%) of phages KP27, KP16, KP36, KP32 and KP34 adsorbed to *K. pneumoniae* cells within 5 min. By comparison, only 75% of KP15 adsorbed within this time span. The one-step growth curve indicated that the latent period for *Siphoviridae* and *Podoviridae* viruses was short (15 min) and the estimated burst size was ~50-60 phage particles per infected bacterium. The *Myoviridae* representatives multiplied more slowly (25 min latent period) and with lower burst size (10–15 PFU per infected cell).

The *Podoviridae* representatives and KP15 were shown to be relatively sensitive to high temperature, with a 100-fold decrease in titre observed after 10 min at 60°C. The concentration of PFU/ml of KP27, KP16 and KP36 was reduced by 0.5-1 orders of magnitude. With the possible exception of phage KP15, which suffered a 1.5 order decrease of virion numbers, the remainder of the phages were unaffected by chloroform. The susceptibility of KP15 (T4-like phage) to chloroform could be explained by the observation that despite the general absence of lipids, about one third of tailed phages are chloroform-sensitive [23].

The susceptibility to different pH conditions showed that phage particles were relatively stable within a pH range of 5–8. Incubation at pH 4 caused a 3-log decrease in phage titre of KP16 and KP34, while the other phages displayed greater stability at this pH.

### Restriction analysis of phage DNA

**Figure 3:**
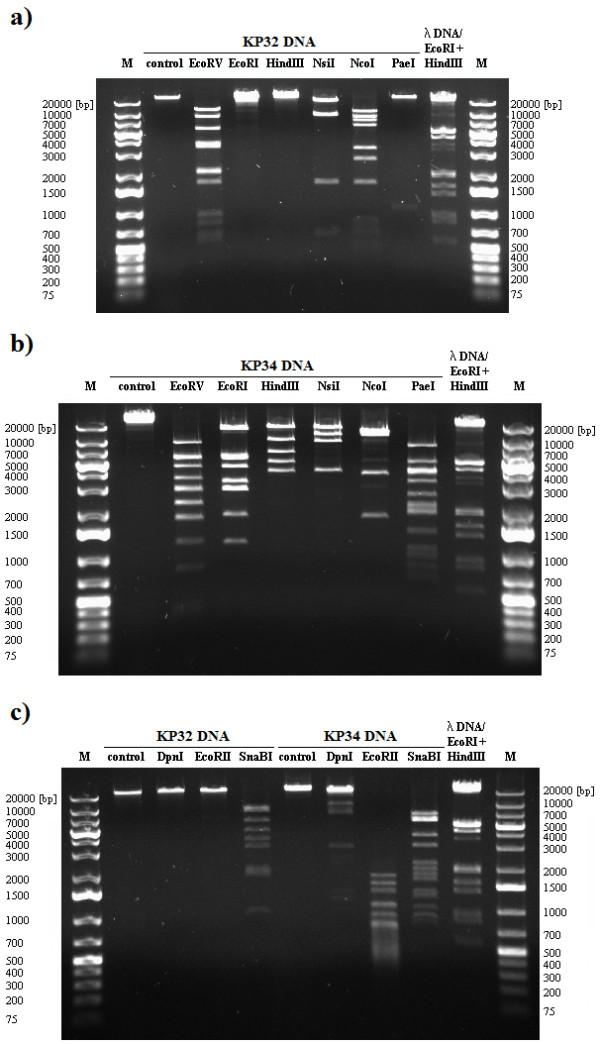
**DNA restriction endonuclease digestion of KP32 (a, c) and KP34 (b, c) from *Podoviridae *family.**

**Figure 4:**
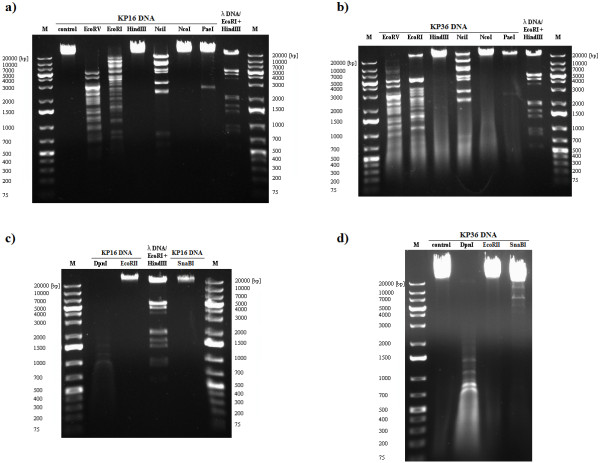
**DNA restriction endonuclease digestion of KP16 (a, c) and KP36 (b, d) from *Siphoviridae *family.**

**Figure 5:**
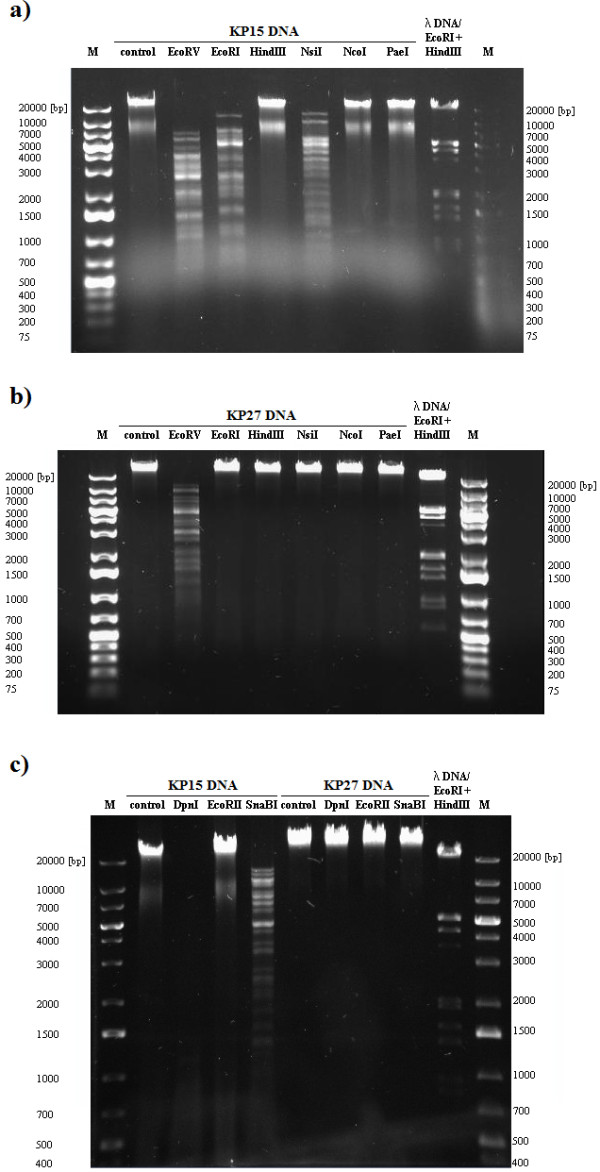
**DNA restriction endonuclease digestion of KP15 (a, c) and KP27 (b, c) from *Myoviridae *family.**

The DNA restriction patterns were compared to *in silico* genome digestion analysis of selected phages. Genomes of five phages have been previously sequenced and deposited at GenBank, namely KP32 [GenBank: GQ413937], KP34 [GQ413938], KP15 [GU295964], KP27 [HQ918180], and KP36 [JQ267364]. Phage DNA was digested with restriction endonucleases sensitive to prokaryotic DNA methylation: EcoRV, EcoRI, HindIII, NsiI, NcoI, PaeI, and SnaBI. These are also insensitive to Dam and Dcm modification of DNA: EcoRII, which is blocked by Dcm methylated DNA, and DpnI endonuclease, which cleaves only Dam methylated DNA (Figure 3–5).

The DNA of podovirus KP34 was sensitive to all restriction enzymes (Figure 3b, c) including DpnI, indicating DNA modification of GATC by Dam methyltransferase. Since the KP34 genome lacks a methyltransferase-encoding gene, the modification must be a result of the host enzyme [24].

In contrast, the second representative of the *Podoviridae* family, phage KP32, was highly refractory to digestion by endonucleases, including EcoRI, HindIII, DpnI, and EcoRII (Figure 3a, c). The *in silico* analysis revealed that the DNA lacked target sequences recognized by EcoRI and HindIII. Experimental evidence suggests that DpnI cuts the KP32 genome at one site, while *in silico* analysis reveals three cut sites (Webcutter 2.0) suggesting partial modification of GATC sequences as was observed with *Pseudomonas aeruginosa*[25]. Only one EcoRII (CC(A/T)GG) occurs in the DNA sequence of this phage, but this enzyme requires tandem sites for effective cleavage [13]. The difference in sensitivity to restriction endonucleases of above members of the *Podoviridae* provides further evidence that KP32 and KP34 belong to different genera, T7-like virus and φKMV-like virus, respectively, and use different strategies to avoid the host restriction modification (RM) defence mechanisms.

The *Siphoviridae* phage DNA samples (Figure 4) were sensitive to EcoRV, EcoRI, NsiI, and DpnI, and exhibited different but closely related restriction endonuclease patterns. *In silico* digestion of KP36 genome using Webcutter showed 302 cut sites for DpnI and 155 for EcoRII enzymes. DpnI digested KP36 DNA efficiently, but no bands were observed with EcoRII, indicating DNA modification by both Dam and Dcm methyltransferases. The genome sequence of this phage revealed the presence of a DNA adenine methyltransferase (YP_007173578; locus tag G028_gp60) and the lack of cytosine methyltransferases, which again suggested Dcm modification by host enzymes. *Klebsiella pneumoniae* 342 contains both DNA cytosine (YP_002237693) and DNA adenine (YP_002236240) methylases. A similarity in restriction patterns between both *Siphoviridae* phages (KP16 and KP36) indicates the presence of the same mechanisms of resistance to bacterial RM systems.

The agarose gel electrophoresis banding patterns obtained after restriction enzyme digestion of *Myoviridae* phage DNA confirmed that they were genetically different (Figure 5). KP15 DNA was resistant to digestion by several endonucleases, including HindIII, NcoI, PaeI, and EcoRII (Figure 5a, c). The *in silico* analysis of DNA cleavage revealed no sequences recognized by HindIII, NcoI and PaeI enzymes. The restriction resistance profiles indicated that KP15 phage DNA could have numerous modifications described in other T4-like bacteriophages. Almost total degradation of DNA by DpnI enzyme and 1148 cut sites found (Webcutter 2.0) suggested adenine methylation modification. In the case of EcoRII, 279 recognition sequences were detected *in silico* but no digestion was observed in gel analysis, which is evidence of Dcm methylation. Both Dam and Dcm methyltransferase genes were found in the KP15 genome.

Phage KP27 DNA was highly resistant to restriction digestion, and among the nine endonucleases tested only EcoRV showed activity (Figure 5b, c). *In silico* analysis showed that the DNA lacked HindIII, NcoI and PaeI sites. While the DNA contained numerous EcoRI (73), NsiI (66) and SnaBI (40) recognition sequences, it was resistant to these endonucleases. In the case of EcoRII, 276 recognition sequences were detected *in silico*, but no digestion was observed in gel analysis, offering evidence of Dcm methylation, which is encoded by the KP27 genome (AEX26604; locus tag KP27_137). The interesting results were obtained subsequent to attempted DpnI cleavage. While almost total degradation of DNA by DpnI enzyme was expected due to the presence of 1130 cut sites and the phage-encoded Dam methyltransferase gene, no digestion was observed. Comparing KP15 and KP27 DNA restriction patterns *in silico* illustrates similar sensitivity to experimental endonuclease activity and similar rates of target sites were obtained. Digestion profiles were repeated, with KP15 and KP27 phages propagated on *K. oxytoca* instead of *K. pneumoniae*. The same *K. oxytoca* strain was used for both phages to avoid differences in the host RM system and identical restriction patterns were obtained (data not shown). Generally, the restriction endonuclease digestion results revealed that all *Klebsiella* phage DNAs were sensitive to EcoRV activity regardless of genetic differences and modifications.

### Discussion and conclusions

The *Klebsiella* bacteriophages described in recent papers belong to *Podoviridae*, *Myoviridae* and *Siphoviridae* families [26-30]. During this study 32 different viruses from all the aforementioned taxonomic groups were isolated and characterised. Members of the *Myoviridae* exhibited the broadest spectrum of lytic activity. These viruses replicated on *K. pneumoniae* strains but also showed lytic potential against *K. oxytoca* strains. No correlation was found between phage sensitivity and bacterial ESBL enzyme production. Generally, the *Podoviridae* and *Siphoviridae* viruses exhibited a narrower lytic spectrum of activity but multiplied more efficiently and elicited faster elimination of the bacterial host. The lytic efficiency of these viruses was enhanced by the production of phage-encoded soluble enzymes, including polysaccharide depolymerases illustrated as a plaque halo [31]. Depolymerases increase antibacterial activity by disruption of the *Klebsiella* capsule, rendering the cells more susceptible to antibacterial agents such as other phages, antibiotics, and the immune system.

The genome sequence of phage KP36 reveals that it is a member of the *Tunalikevirus* genus. Since KP16 displays a restriction digestion profile very similar to that of KP36, and infects a similar number of strains, we believe that they both belong to this genus. KP36 appears to have evolved several mechanisms for evading host restriction. The low incidence of restriction sites for HindIII, NsiI, NcoI, PaeI, and SnaBI suggests counter selection of these sequences similar to the strategy of T7-like virus. Interestingly, its genome contains five sites for the type I restriction endonuclease KpnA1 (GAA(N6)TGCC) and two for KpnB1 (CAAA(N6)RTCA). Infection by coliphage T1 results in enhanced expression of adenine methyltransferase [32,33], and increased N6-methyladenine in its genome [34]. Since phage KP36 encodes a similar enzyme, we would expect a similar phenotype.

An example of perfect adaptation to various RM systems by loss of restriction sites is the T7-like podovirus KP32, whose DNA contains a low number of sites for all the tested restriction endonucleases. In addition, its genome contains three KpnA1 and two KpnB1 sites. Such a strategy presumably allows this virus to propagate on diverse *Klebsiella* strains and is a positive selectable trait for this phage group. By comparison, the second member of the *Podoviridae* family – the φKMV-like phage KP34 – was susceptible to all endonucleases but also exhibited Dam methylation in spite of the fact that this virus lacks its own methyltransferase gene. It is proposed that phage KP34 may use a mechanism similar to lambdoid phages to stimulate host methyltransferase activity.

The restriction resistance profiles of myoviruses KP15 and KP27 indicate that the DNA of these phages probably possesses T4-like mechanisms to overcome the RM systems of potential bacterial hosts. *In silico* and *in vitro* analyses of KP15 DNA confirmed the presence of virus-encoded Dam and Dcm methyltransferases. There was no evidence that these phages synthesized atypical nucleotides (hmC or glucosyl hmC), as indicated by the lack of dCMP hydroxymethyltransferase and glucosyltransferase genes. Surprisingly, the virus-encoded Dam methyltransferase did not efficiently modify KP27 DNA.

Phages as important tools in the elimination and clinical alleviation of *Klebsiella* colonization have been illustrated by the recent papers of Hung *et al.*, Karamoddini *et al.* and Kumari *et al.*[35-39]. In selection for safe antimicrobials, the comprehensive characterization of the genome for unfavourable genes such as lysogeny-associated genes and toxins is essential alongside biological, physico-chemical and phenotype characterisation [40-42]. Characterisation of phages enables a better understanding of how their biology, including host specificity, adaptation to bacterial defence systems and propagation dynamics in natural systems, will assist in programmes to exploit bacteriophages as therapeutic agents against bacterial pathogens. In this study the phenotypic features and genetic properties of six *Klebsiella* phages have been examined and could be used as basic knowledge for future therapeutic work.

## Materials and methods

### Origin of bacteriophages

Ten clinical *Klebsiella pneumoniae* ESBL(+) strains plus ATCC 700603 were used as hosts for bacteriophage isolation and propagation from the eight water samples. The samples were taken from different environmental sources (Table 1). All samples were examined for faecal contamination using enteric viral pathogen assay. The *Escherichia coli* Famp (ATCC 700891) strain was applied as the host for male-specific (F^+^) coliphages (FRNAPH) and *Escherichia coli* B as an indicator of the somatic coliphages (SOMCPH) propagation [43]. The bacteriophage titre in the water sample was assessed using the double-agar layer technique as according to methods described previously [44].

### Isolation of phages

A water sample was centrifuged (15 000 × g/15 min) and the supernatant filtered through a 0.22 μm Millex-GP filter. Phage propagation followed the method of Ślopek *et al.*[45]. One milliliter of filtered water sample and 0.5 ml of *K. pneumoniae* ESBL (+) host strain, grown overnight in Mueller Hinton Broth (MHB) (bioMérieux Polska, Warsaw, Poland) were added to 10 ml of MHB and incubated at 37°C until complete lysis appeared (approximately 4–6 h). The suspension was then filtered through a 0.22 μm Millex-GP filter. The procedure was repeated thrice to eliminate bactericidal activity of some chemical compounds present in the water samples. The bacteriophage titre in the filtrate was assessed as described above [44].

### Electron microscopy

A high-titre phage lysate previously filtered through a 0.22 μm Millex-GP filter was centrifuged at 25 000 × g for 60 min and the pellet was washed twice in 0.1 M ammonium acetate, pH 7.0. A sample (10 μl) of the resuspended sediment was deposited on carbon-coated Formvar films, stained with 2% uranyl acetate and examined in the transmission electron microscope (TEM) JEM-100C (JEOL LTD, Tokyo, Japan) at 80 kV with magnification of 66 000×. The phage size was determined from the average of 5–7 independent measurements using T4 phage tail (114 nm) as the magnification control.

### Determination of phage host range

The strains used in this study are listed in Table 2. Bacteria were stored at −70°C in Tryptone Soy Broth (TSB; Becton Dickinson and Company, Cockeysville, MD) supplemented with 20% glycerol. Prior to phage sensitivity testing bacteria were subcultured in TSB. For all phage experiments 4–6 h bacterial growth, unless otherwise stated, was used. To determine bacterial susceptibility to phage-mediated lysis, bacteria grown on liquid MHB medium were transferred directly onto MHA agar plates (bioMérieux). After drying, a drop of the phage suspension (10^8^ PFU/ml) was put on the bacterial layer and incubated at 37°C. The plates were checked 4–6 h and again 18 h later for the presence of bacterial lysis. Spot testing is the rapid and efficient method for determining the host range in large collection of bacteria [46].

### Phage adsorption procedure

The adsorption of phages to bacterial host cells was examined using a slight modification of previously described methods [45,47]. Cells from an overnight culture plate (MHA) were suspended in MHB to OD_600_ ~0.9-1.0. An equal volume of bacterial suspension and phage diluted to 10^5^-10^6^ PFU/ml (MOI 0.001) were incubated at 37°C for 5 min. After incubation the culture was filtered (0.22 μm) and the free phages enumerated, in triplicate, in the filtrate using the double-agar-layer method. The reduction in phage titre was the number of phages adsorbed to the cells. No reduction in phage titre in control filtration (0.22 μm Millex-GP filters) was observed.

### Burst size experiments

A one-step growth curve of isolated *Klebsiella* phages was performed according to the method of Pajunen *et al.*[48] with modifications. The density of a mid-exponential bacterial culture in MHB was adjusted to 2 × 10^8^ CFU/ml. To 0.9 ml of this cell suspension was added 0.1 ml of bacteriophage in order to achieve a multiplicity of infection of 0.005. Phages were allowed to adsorb for 5 min at 37°C, after which time the mixture was diluted to 10^-5^ and samples, in triplicate, were taken at 5 min intervals for titration. Experiment was performed at three different times and, the values depict the mean of three observations ± standard deviation (SD).

### Sensitivity of phage particles to temperature, chloroform and pH

An equal volume of filter-sterilized bacteriophage (10^7^ PFU/ml) was mixed with chloroform and incubated for 2 h at room temperature with intermittent shaking. Further preparations of phages were incubated at pH 4, 5, 6 and 8 for 1 h at room temperature. A phage preparation was also incubated at 60°C for 10 min. After all these experiments the bacteriophage titre was assessed using the double agar layer technique [44].

### Isolation and restriction digestion of phage DNA

The DNA of six bacteriophage isolates (KP15, KP16, KP27, KP32, KP34 and KP36) were extracted and purified from phage lysates using a QIAGEN® Lambda Midi Kit (QIAGEN Inc., Valencia, CA, USA) and following the manufacturer’s protocol. These preparations were digested with restriction endonucleases which differed in sensitivity to DNA methylation (EcoRV, EcoRI, HindIII, NsiI, NcoI, PaeI, DpnI, EcoRII, SnaBI) purchased from Fermentas Life Science (Vilnius, Lithuania). The description of each restriction enzyme used in the study is presented in Table S1 (see Additional file 1: Table S1).

After the enzymatic digestion, the DNA fragments were separated by electrophoresis in a 0.6% agarose gel containing ethidium bromide (0.5 μg/ml) in Tris-boric acid-EDTA buffer, at 90 V in a Bio-Rad agarose gel electrophoresis system (Bio-Rad Laboratories, Inc., Hercules, CA, USA). GeneRuler™ 1 kb Plus DNA Ladder (Fermentas Life Science) was used as a size marker. Restriction digestions were carried out in triplicate. The restriction patterns were compared with *in silico* genome digestion done with Webcutter 2.0 (http://rna.lundberg.gu.se/cutter2/).

## Competing interests

The authors declare that they have no competing interests.

## Authors’ contributions

AKS carried out the phage isolation and multiplication, plaque assays, phage DNA extraction, restriction map analysis and helped to draft the manuscript. JK carried out the transmission electron microscopy analysis. AKS, BWD, MLS determined of phage host range. MLS, MZ performed sensitivity of phage particles to temperature, chloroform and pH. BWD, DA, GMS carried out phage adsorption procedure and burst size experiments. ZDK, AG provided materials and equipment. ZDK, AMK analysed the data and prepared manuscript. All authors read and approved the final manuscript.

## Supplementary Material

Additional file 1: Table S1 The description of each restriction enzyme used in the study. Click here for file
