# Supplementary material for: Characterising the biology of novel lytic bacteriophages infecting multidrug resistant Klebsiella pneumoniae
Source: Virol J. 2013 Mar 28;10:100. doi: 10.1186/1743-422X-10-100 (PMC3620542; doi:10.1186/1743-422X-10-100)
Supplement: Additional file 1: Table S1 — The description of each restriction enzyme used in the study. [file 1743-422X-10-100-S1.pdf]

Table S1. The description of each restriction enzyme used in the study.

**Enzyme**	**Sequence**	**Methylation effects**
EcoRV	5'...□GAT↓ATC...3'	No methylation effects on digestion
EcoRI	5'...G↓AATTC...3'	No methylation effects on digestion
HindIII	5'...□A↓AGCTT... 3'	No methylation effects on digestion
NsiI	5'...□ATGCA↓T...3'	No methylation effects on digestion
NcoI	5'...C↓CATGG…3'	No methylation effects on digestion
PaeI	5'...□GCATG↓C...3'	No methylation effects on digestion
DpnI	5'...G^{m}6A↓TC...3'	Does not cut *dam*$^{-}$ DNA
EcoRII	5'...□↓CCWGG...3'	Dcm: completely overlaps – blocked
SnaBI	5'...TAC↓GTA...3'	CpG: completely overlaps – blocked
